# Partial glossectomy for treating extensive oral squamous cell papilloma^☆^

**DOI:** 10.1016/j.bjorl.2016.07.002

**Published:** 2016-08-10

**Authors:** Emerson Filipe de Carvalho Nogueira, Pedro Henrique de Souza Lopes, Bruno Luiz Menezes de Souza, Cleice Barbosa Bezerra, Ricardo José de Holanda Vasconcellos, Belmino Carlos Amaral Torres

**Affiliations:** a Universidade de Pernambuco (UPE), Faculdade de Odontologia de Pernambuco, Camaragibe, PE, Brazil; b Hospital Regional do Agreste, Caruaru, PE, Brazil; c Universidade Estadual da Paraíba (UEP), Campina Grande, PB, Brazil

## Introduction

Squamous cell papilloma is a benign proliferation of the stratified squamous epithelium, which results in a papillary or warty mass, which is presumably induced by the human papilloma virus (HPV).^1^ Currently, there are at least 24 types of HPV associated with head and neck lesions; the pathogenesis of squamous papilloma is related to HPV types 6 and 11.^2^ The virulence and infectivity of oral papilloma is extremely low, unlike other HPV-induced lesions.^1^

Clinically, oral papillomas usually present as an exophytic, isolated, pedunculated, painless growth of less than 1 cm, the surface of which shows fingerlike or warty projections resembling a cauliflower, exhibiting color ranging from pink to pale white, depending on the degree of associated keratinization. They are more frequently found in the tongue and palate, affecting both genders equally and, commonly, individuals between 30 and 50 years.^3^ The differential diagnosis includes verruca vulgaris, focal epithelial hyperplasia and condyloma.^4^ Warty lesions in the oral cavity that are generated by viral infection have quite similar characteristics, with histopathological analysis necessary for diagnotic confirmation.^5^

The treatment for oral papilloma is conservative, requiring complete removal of the lesion, but excision of a surrounding margin of safety is not mandatory. Untreated lesions usually do not change over time. Conservative surgical excision is a good choice, with the destruction by CO_2_ laser, cauterization or cryosurgery also being acceptable.^6^

Although the squamous papilloma normally presents with small dimensions, some cases develop and reach larger sizes. The purpose of this paper is to report a rare case of extensive oral squamous papilloma with involvement of the tongue, treated by partial glossectomy.

## Case report

**Figure 1:**
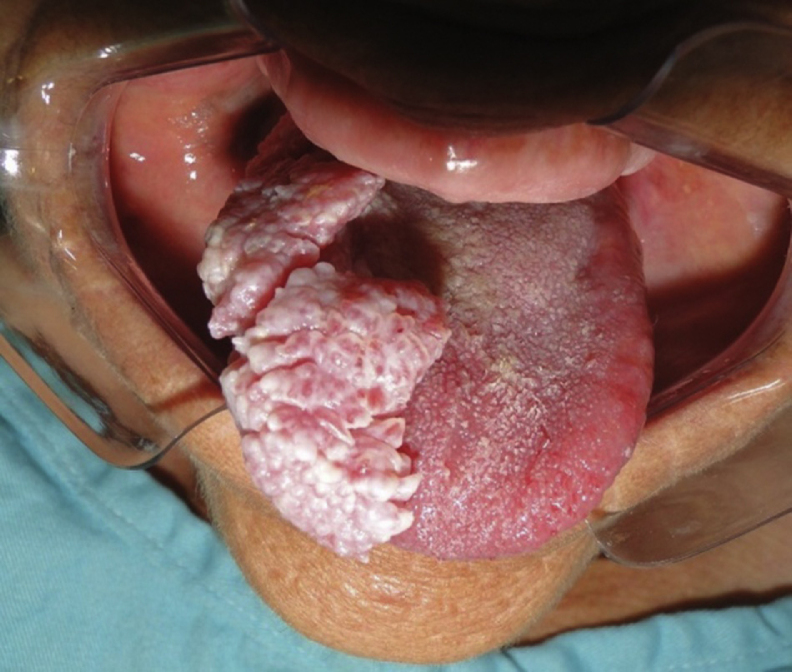
Warty aspect of the lesion on the dorsum and lateral border of the tongue.

**Figure 2:**
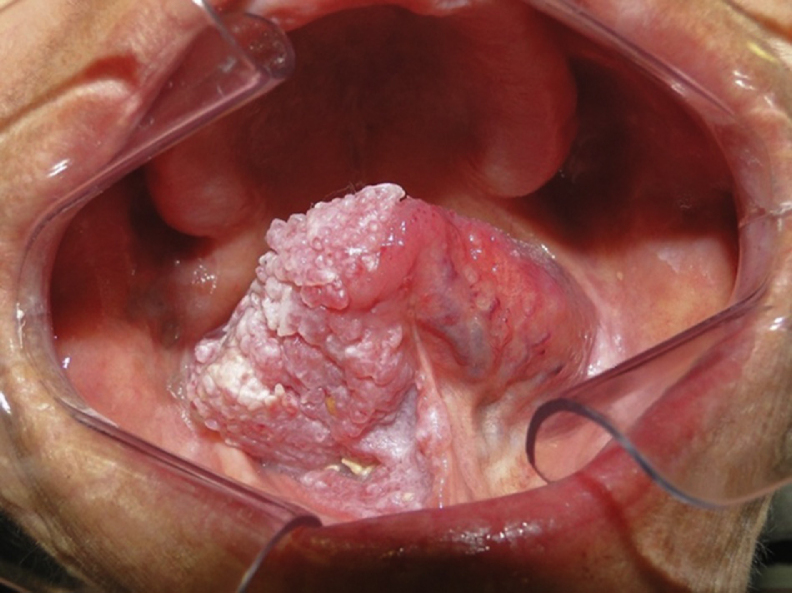
Lesion involvement on the ventral surface of the tongue and part of the buccal floor.

A 73-year-old female patient presented with complaints of an enlargement and change of consistency of the tongue over the prior 15 years. The patient denied any systemic disease or medication of continuous use. Extra oral physical examination including cervical palpation revealed no noteworthy abnormalities. The intra-oral physical examination showed a vegetative lesion of verrucous aspect involving virtually the entire right portion of the tongue, extending from the lingual apex to the posterior region, close to vallate papillae, being approximately 6 cm in its greatest length (Figure 1, Figure 2), but with no involvement of the submandibular gland duct. On palpation, it had a softened and velvety consistency; the color was similar to the lingual mucosa with some more pale areas. The hematologic preoperative examination consisted of ELISA research for HIV, which was negative.

**Figure 3:**
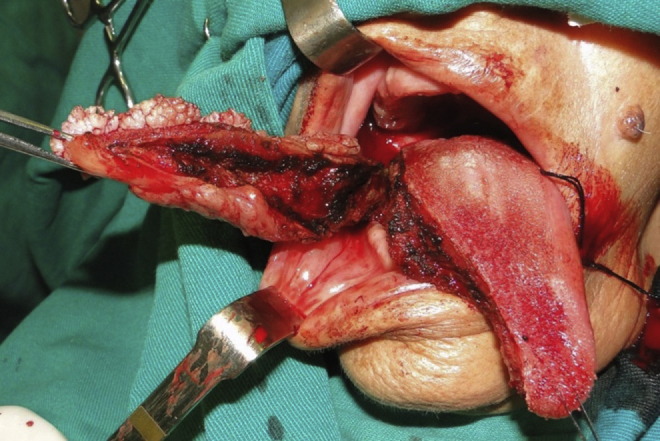
Partial glossectomy.

**Figure 4:**
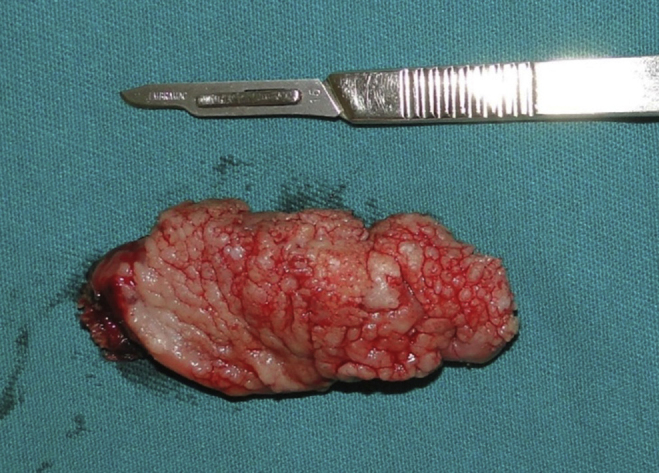
Surgical piece.

Under local anesthesia, incisional biopsy was performed on various regions to minimize the risk of an underdiagnosis of a malignant lesion, which confirmed a diagnosis of squamous papilloma. Thus, we opted for surgical treatment to completely remove the lesion by partial glossectomy. Under general anesthesia, a needled 2.0 cotton suture was placed at the lingual apex region for better traction and lingual repair. This way, an atraumatic resection of the lesion was accomplished with the use of electrocautery (Figure 3, Figure 4), preserving the duct of the submandibular gland, and subsequently suturing the surgical wound margins with Vicryl 3.0 suture. A nasoenteric tube was installed for nutrition during the postoperative healing period, in order to avoid possible infections, and provide greater comfort for the patient.

The definitive histopathological examination confirmed the initial diagnosis, and was characterized by parakeratosis, acanthosis, papillomatosis, mild atypical cells, and white blood cell exocytosis; chorion with hyperemia, foci of hemorrhage, and mild diffuse inflammatory infiltrate of mononuclear cells and eosinophils.

At the follow-up visit, points of dehiscence were observed postoperatively, which were treated with strict oral hygiene, and healed with no signs of infection during the whole period of tissue repair, with adequate formation of granulation scar tissue on the site. After 10 days, the nasoenteric tube was removed.

**Figure 5:**
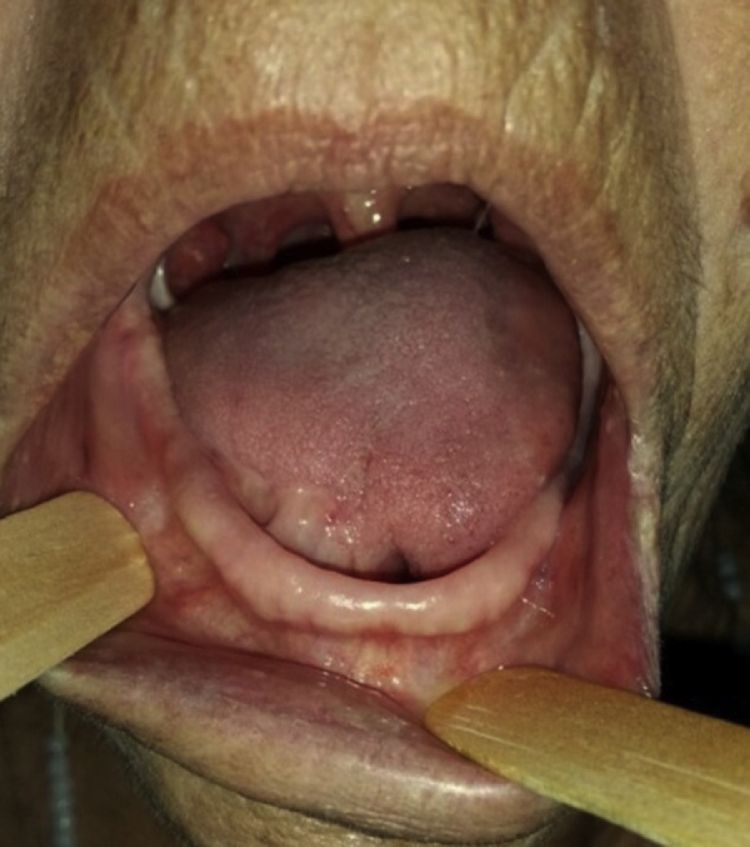
Post-operative period of 20 months.

The patient has been followed in an outpatient clinic for 20 months with no signs of recurrence (Fig. 5). From the phonetic and nutritional points of view, the patient had adequate speech therapy follow-up, allowing satisfactory recovery of speech and swallowing ability.

## Discussion

Oral papillomas are usually clinically asymptomatic, exophytic, and have a cauliflower-like surface. They are most commonly found in the tongue, lips, buccal mucosa, gums, palate, and their size range from 0.2 to 1 cm. Authors such as Kumar et al.^3^ and Martins Filho et al.^5^ observed dimensions that are similar to that found in the literature. Evolution time is variable, and lesions can be observed with onset from a few weeks up to 20 years.^7^, ^8^ In this case, the patient's lesion developed over about 15 years.

The occurrence of such lesions is influenced by smoking, coexisting infections, nutritional deficiencies, hormonal changes and immunological changes, such as in cases of HIV-positive patients, who are generally affected by multiple oral lesions.^3^, ^9^ Squamous papillomas are traditionally divided into two types: isolated-solitary and multiple-recurring. The former is generally found in the oral cavity of adults, while the latter is mainly found in the children with laryngotracheobronchic complex.^2^ In the present case, the solitary-isolated type was observed, and no systemic alteration or predisposing factor was found.

Carneiro et al.^7^ conducted a clinical and histopathological study of 12 patients with suspected squamous papilloma. The most prevalent site was the tongue (41.7%), followed by the palate (33.3%), lip (16.7%) and labial commissure (8.3%), and the size of the lesions varied from 0.2 to 1.2 cm. Abbey et al.^8^ analyzed 464 papillomas of oral squamous cells, and found that lesions were more frequent on the palate when compared to other regions, such as lateral edge of the tongue and lips. Lesion size was recorded in 141 cases where 107 (75.9%) were smaller than 1 cm, and 34 (24.1%) were larger than 1 cm, and among these, the maximum size found was 3 cm. In the present case, the lesion had very extensive dimensions, not corroborating the findings reported in the world literature.

Similar to this study, Martins Filho et al.^5^ and Jaju et al.^9^ histologically observed the presence of parakeratinized epithelial lining of stratified squamous type, acanthosis, papillomatosis, and koilocytosis, thus confirming the definitive diagnosis. In this case, mild cellular atypia was also seen, a finding that further suggests surgical removal. However, this is a discrete finding that supports lesion excision, rules out malignancy and is conclusive for oral papilloma. Since we concluded from the clinical and histopathological examinations that we would perform extensive resection of the lesion, we opted to not perform viral typing, since it is costly, performed only in large cities, and would not alter our surgical planning.

Although the definitive treatment under local anesthesia is the most common procedure^2^ and gives satisfactory results, this technique was not recommended for the reported case. We opted for general anesthesia due to the size of the lesion, greater safety and comfort to the patient regarding possible complications, and maintenance of vital signs.

The literature suggests several modalities of treatment for oral squamous papilloma. Among these are conventional surgical excision, cryosurgery, laser ablation, intralesional injection of interferon, and application of salicylic acid.^10^ The conservative surgical removal involving the base of the lesion is the gold standard for the treatment of this disease, with low risk of recurrence reported in the literature.^1^, ^3^, ^9^ In this case, we opted for surgical removal with conservative margins, allowing the achievement of satisfactory functional results in long-term monitoring, and absence of signs of recurrence.

## Conclusions

As the papilloma is usually asymptomatic lesion, and usually presents with small dimensions, it is usually treated with surgical removal during excisional biopsy. However, some cases are larger and cause discomfort, difficulty in speech and chewing, social impairment, and their treatment can result in greater deformity after the final treatment.

## Conflicts of interest

The authors declare no conflicts of interest.
